# Improving treatment outcomes for adolescents with borderline personality disorder through a socioecological approach

**DOI:** 10.1186/s40479-022-00187-9

**Published:** 2022-06-15

**Authors:** Sune Bo, Carla Sharp, Mickey T. Kongerslev, Patrick Luyten, Peter Fonagy

**Affiliations:** 1 Department of Psychology, University of Copenhagen, Copenhagen, Denmark; 2 Child and Adolescent Mental Health Services, Ny Oestergade 12, 4000 Roskilde, Region Zealand Denmark; 3 Department of Psychology, University of Houston, Houston, USA; 4 Department of Psychology, University of Southern Denmark, Odense, Denmark; 5 Faculty of Psychology and Educational Sciences, University of Leuven, Louvain, Belgium; 6 Research Department of Clinical, Educational and Health Psychology, University College London, London, UK; 7 Anna Freud Centre, London, UK

**Keywords:** Personality disorders, Adolescent, BPD, Treatment, Socioecological treatment, Epistemic trust, Mentalization-based treatment, Psychotherapy research

## Abstract

**Background:**

There is a dearth of studies evaluating treatment efficacy for adolescents diagnosed with borderline personality disorder. The few available randomized controlled trials that have been conducted show modest results and treatments appear to have equivalent effects. The current paper draws on (a) the lessons learnt from the last 50 years of psychotherapy research in general and (b) recent advances in mentalization-based understanding of why treatment works, which together point to the importance of following a socioecological approach in the treatment of personality problems in adolescence – a developmental period that insists on a treatment approach that goes beyond the therapist-client dyad.

**Case presentation:**

Here, we describe such an approach, and offer a clinical case example with a young 16-year old girl diagnosed with borderline personality disorder, to illustrate what a shift toward a more socioecological approach would entail.

**Conclusions:**

The clinical impact of the socioecological approach and the potential benefits as illustrated in the current case illustration, offers a framework that justifies and allows for the expansion of service delivery for youth with borderline personality disorder beyond dyadic therapist-client work.

## Background

Whilst research supporting the effectiveness of a wide range of relatively different psychotherapeutic treatments for adults with borderline personality disorder (BPD) is growing, there is still a relative dearth of studies concerning the effectiveness of treatments for adolescents diagnosed with BPD. This, yet nascent state of affairs offers the opportunity to be more intentional in the design and planning of research on treatment programs with adolescents with BPD. In this paper, we suggest a framework for developing and evaluating treatment programs for adolescents with BPD by drawing on the lessons learnt from three sources: the last 50 years of (a) adult and (b) child and adolescent’s psychotherapy research and (c) the mentalization-based approach to BPD. We start by reviewing the current evidence-base for psychotherapeutic treatment of BPD in young people to illustrate the need for innovation. Next, we present how recent advances in mentalization-based theory can explain the lessons learnt from general psychotherapy research and present a broader socioecological approach to the treatment of BPD in young people, drawing on mentalization-based theory. Finally, we illustrate the socioecological approach with a case-example of an adolescent diagnosed with BPD, to elucidate the different interventions suggested in this treatment approach.

### Treatment of BPD in adolescence: What do we know?

The last 15 years of research conclude that BPD in adolescence is (a) a valid and reliable diagnosis [1], (b) shows the same or even higher prevalence rates as in adult populations [2], (c) is associated with a marked decrease in social functioning [3], (d) is linked to individual suffering [4], (e) demonstrates high levels of comorbidity [5], and (f) incurs a substantial financial burden to both the educational as well as the mental health systems [6]. Nonetheless, there is a paucity of evidence on the efficacy of treatment for BPD in adolescents, despite the growing evidence for the effectiveness of psychotherapy in adults with BPD [7].

Only nine RCTs have been conducted evaluating the efficacy of specialized treatment programs in adolescents with BPD [8]. While these studies have consistently shown that psychotherapy may be effective in the treatment of BPD features in young people, only in one trial [9], was the active treatment arm superior to control treatment in terms of reducing BPD pathology. In a recent systematic review and meta-analysis of psychotherapies for BPD in adolescents, it was found that psychotherapy significantly reduced BPD pathology and self-harm, but that these effects were somewhat fleeting and disappeared at follow-up when compared to control treatments [10]. Thus, the effects of BPD interventions in adolescence appear to be modest, inflated by risk of bias, and particularly unstable at follow-up [8]. In fact, a recent Cochrane review on the effectiveness of psychotherapeutic treatments for BPD found that the effect of treatment was lower for people aged younger than 18 years of age compared to those above [7]. Accordingly, despite trends pointing to a small but growing variety of promising psychotherapeutic treatment programs for adolescents with BPD, the available research identifies many important gaps in our knowledge and reveals that the treatments are not as effective as we would like them to be [11, 12]. Thus, with limited results regarding treatment of adolescents with BPD, we lack knowledge to decide how to best design a treatment approach for those young people. In the next section we will review the general psychotherapy literature and search for clues that can guide which factors to incorporate in a treatment approach for adolescents with BPD that may enlarge effect sizes in treatment outcomes.

### What works in psychotherapy? The role of general factors

Findings that no single specific psychotherapeutic treatment approach appears to show superiority in adolescent BPD mirrors the now rather widely accepted finding in adult samples that all *bona fide* types of psychotherapy seem to be equally effective [13]. The similarity here naturally invites us to draw on what is known in adult psychotherapy research to potentially sidestep important pitfalls that have slowed down progress in adult psychotherapy research in our goal to improve treatment effectiveness for adolescents with BPD. The following set of findings seem to be important in thinking about the future development of treatments for BPD in young people: (1) the efficacy of psychotherapy in general, (2) the role of common factors, (3) therapist characteristics, (4) placebo effects, (5) feedback informed therapy, (6) manualized vs non-manualized psychotherapy, and (7) extra-therapeutic factors.

### The efficacy of psychotherapy in general

Approximately 80% to 85% of adults receiving psychotherapy seem to improve when compared to those not in therapy, as reflected in a large and robust effect size (ES) of 0.85 [14]. For youth, the mean post-treatment effect size has been shown to be substantially lower (ES = 0.45), particularly in those with complex psychological problems [15, 16]. Moreover, psychotherapy research in both adults [17] and in children and adolescents [18] have shown but little improvement in terms of relative effectiveness, reporting primarily modest effect sizes over the last four decades, despite an increase in the number of specialized treatments. Also, a recent comprehensive multilevel meta-analysis spanning 53 years (1963–2016), including 453 RCTs and 31,933 patients between 4–18 years, found that the mean effect size for improvement in outcomes increased non-significantly for anxiety disorders, decreased non-significantly for ADHD, and decreased significantly for depression and conduct problems [19]. Further, one study exploring outcomes of children being treated in either a community mental health care service (*N* = 936) or in a managed care setting (*N* = 3,075) found the deterioration rates in these settings to be 24% and 14%, respectively [20]. Hence, general findings concerning the effectiveness of psychotherapy seem to be mirrored in current findings concerning the effectiveness of psychotherapy in young people with BPD and should lead us to pause and reflect whether we are skating to where the puck is going to be.

### The role of common factors and the therapeutic alliance

Even in the presence of positive therapeutic change, it remains unclear *how* therapy works, resulting in a paucity of evidence-based explanations of *how* or *why* even the most well-studied intervention programs generate change [21].

Arguably, the current leading common factor model is the contextual model proposed by Wampold and Imel [13] formulated as an alternative to the medical model in which psychotherapy effects are thought to mainly result from specific interventions tailored to specific disorders. Wampold and Imel’s contextual model is a common factor model, which in contrast to the medical model, suggests that psychotherapy works through three pathways: (1) a genuine relationship, (2) expectation and (3) specific therapeutic ingredients (common in all specific psychotherapies) that foster hope [22].

The most researched common factor is that of the therapeutic alliance. In a meta-analysis including 295 studies comprising more than 30,000 patients the overall alliance–outcome association for psychotherapy was *r* = 0.28, equivalent to a medium effect size of Cohen’s *d* = 0.58 [23]. These results confirm the robust positive relationship between alliance and outcome across alliance and outcome measures, cultures, patient characteristics, treatment approach, and the perspective of the assessor. This finding has also been confirmed applying instrumental variable analyses [24]. Although the therapeutic relationship has a long tradition in child and adolescent psychotherapy dating back to Anna Freud [25], there is a scarcity of research on the alliance-outcome relationship in child and adolescent psychotherapy [26]. Results from a recent meta-analysis including only prospective studies in young people revealed a weighted random effect size of *r* = 0.19 (*k* = 28, *N* = 2,419, *p* < 0.01), indicating a small to medium effect, equivalent to *d* = 0.39 [27]. Another study, focusing on adolescents in mental health treatment, and controlling for treatment-level confounds and extracting individual effect sizes using a preregistered conceptual hierarchy, revealed an effect size of *r* = 0.29, *k* = 28, *N* = 2911, *p* < 0.0001 [28]. A reason for the apparently lower effect size of the alliance in adolescent samples as compared to adults could be that young people often enter into psychotherapy because other people such as their parents want them to, presumably making the task of developing a strong alliance more difficult [29]. Indeed, research indicates that the alliance between the parent/caregiver and the therapist is as closely related to positive outcomes as the alliance between the therapist and the adolescent [27], underscoring the importance of engaging parent/caregiver actively in the treatment, not the least in the case of working with adolescents.

### The role of therapist characteristics

Available data suggest that therapist characteristics seems to be an important, but also historically overlooked determinant of outcome in psychotherapy, explaining between 5 to 9% of the variance in outcome [30, 31]. This appears as a relatively small percentage, but in most meta-analyses, psychotherapy overall compared to waiting-list control, account for only 20% of the variance in outcome, encompassing all aspects of psychotherapy including specific factors, common factors, therapist factors, alliance, adherence etc. [32]. Hence, therapist characteristics account for a relatively large proportion of the known variance in outcome. The therapist factor seems to explain more variance than (1) the variability between treatments (0–1%), (2) evidence-based treatments versus placebo (0–4%), and (3) the alliance (5%) [33, 34]. When we ignore the effect of the individual therapist, we are erroneously attributing the effectiveness—or lack thereof—to the specific treatment. One large study with a mixed sample of adults (76%) and adolescents (24%) (*N* = 10,812) treated by 281 therapist showed that outcomes could be improved by referring patients to the most skilled therapists [35]. In another large scale study using multilevel modeling including 10,786 patients and 119 therapists, results clearly indicated large differences in patient outcomes between those therapists recognized as above or below average [36]. Psychotherapy in general is highly effective. But no patient receives psychotherapy in general. Barkham and colleagues report that “the average recovery rate for the more effective therapists is almost twice that of the less effective group” (pp. 22–23) [37]. Specifically, in a study exploring the effect of the alliance on outcome in adolescents with anxiety disorders treated with Cognitive-Behavioral Treatment (CBT), results showed that therapist style, experience and general clinical skills were related to outcome [38]. However, another systematic review and meta-analysis including 1,797 adolescents (*k* = 15) questioned the moderating effect of the therapist characteristics on outcome for adolescent’s psychotherapy [28], rendering the well-established associations between therapist characteristics and outcome reported in adults more uncertain in adolescents populations [39].

### The role of placebo effects

Placebo refers to hope, beliefs and expectations in treatment [40]. Placebo effects have been extensively demonstrated in pharmacological trials [41] as well as in psychotherapy research [42, 43]. Most researchers emphasize that the psychosocial context of placebo effects (i.e., the relationship between the therapist and the patient, the treatment location, variations in cultural beliefs in terms of trusting treatment and the therapist as an authority capable of generating change) most likely constitutes the primary drivers of the placebo response [40].

Placebo effects are rarely studied in child and adolescent populations, and less often in psychiatric settings [44]. Nonetheless, the sparse research available show clear evidence of placebo effects in children and adolescents populations [45], also in psychotherapy trials, including trials in non-psychiatric populations [44].

### Feedback informed therapy

As mentioned earlier, an often-overlooked issue in psychotherapy is to what degree treatment is helpful or not, or perhaps even harmful [20]. Therapists seem to do poorly in predicting their patients outcomes and seem to underestimate poor treatment outcomes in their patients [46]. Therapists also appear to hold overly optimistic views on treatment progress compared to actual measured change [47]. To overcome some of these biases, the implementation of client feedback tools is often used as a method of monitoring patients´ progress and development in therapy, and providing feedback from patients to therapist during treatment [48]. Feedback tools supply therapists with actual, here-and-now information about the therapeutic process supporting therapists´ decision-making through adapting the treatment plan to current issues. This may result in a reduction of treatment failures, and positive effects on psychotherapy outcomes [49]. The American Psychological Association (APA) recommends routine outcome monitoring (ROM) as part of psychological services since it has a positive relationship with outcome in therapy [50]. In a recent meta-analysis including 24 studies, results indicated that in almost two-third of the cases psychotherapy for adults supported by ROM was superior to treatment without ROM [48]. Also, in a study conducted in a multi-center facility for child and adolescent setting, children aged 6–18 with autism spectrum disorder, Feedback Informed Treatment (FIT) was related to enhanced quality of life compared to care as usual [51]. However, in a recent Cochrane review exploring the effect of feedback informed psychotherapy in children and adolescents in mental health settings, no firm conclusion regarding the role of feedback could be reached due to low quality in the included studies [52]. Specifically, for personality disorders (PDs) in adults, one RCT reported adverse effects of feedback informed practice for some types of personality disorders (PDs), namely Cluster B PDs whereas for Cluster C PDs no harmful but rather beneficial effect were observed [53]. So, in terms of implementing FIT therapy in adolescents with BPD, no firm conclusions can be currently drawn, given the small amount of research and lack of actual research on this topic in samples of adolescents with BPD. One could imagine it would be difficult and potentially iatrogenic in youth with BPD, due to complex dynamics which could lead to processes that were split off from the treatment (e.g. punishing the therapist by giving low ratings).

### Manualized vs. non-manualized treatments

The number of manualized psychotherapies has increased dramatically over the last decade and recently APA recognized more than 350 evidence-based psychotherapies based on specific treatment manuals [54, 55].

Specifically for adolescents, some studies support the use of manualized treatment and others question the superiority of manualized treatments as compared to non-manualised interventional strategies [56]. As such, it seems premature to assume that using manuals as such increase effectiveness, though it does appear to increase the internal validity of trials, and, we might speculate, it may also facilitate training. The critique of manualized treatment that tailor specific interventions for specific disorders, has resulted in transdiagnostic [55] and transtheoretical [57] approaches, that extrapolate shared characteristics from various treatment programs to different disorders. The focus is on deducing general treatment strategies and adapting those to the particular treatment context and the particular patient; “*What* treatment, by *whom*, is most effective for this *individual* with that *specific* problem, and under which set of *circumstances*”? as stated in Gordon Paul’s famous quote [58]. Placing the patient in a “diagnostic Procrustean bed” might seem appealing, but it insufficiently considers the variability among patients. Stated otherwise, what works for one type of patient may not work for another one, and “different folks might need different strokes” [59]. Studies suggest that adapting treatment to different patient features such as coping style [60], attachment patterns [61], culture [62], reactance level [63], client preferences [64], and religion [65] may have a positive influence on outcome, emphasizing the need for individualized or tailored interventions. This corresponds with the notion of therapist responsiveness suggested by Stiles (1998), referring to the capacity of the therapist to remain open and flexible and modify interventions to fit the specific needs of the patient and continually modifying those interventions to the state of the patient, the interpersonal and broader social context [57, 66].

### Extra-therapeutic factors

Probably the largest proportion of the variance in psychotherapy outcomes pertains to factors outside of the therapy setting. Originally, Lambert (1992) suggested that extra-therapeutic factors accounted for 40% of the variance in outcome. Subsequent meta-analyses [13] attributed up to 86% of variance in outcomes to extra-therapeutic factors. The key contribution of extra-therapeutic factors to outcomes in psychotherapy for both adolescents and adults is presumably also indicated by findings showing that whether you engage in therapy or not only explains 14% of the variance outcome [31]. Thus, by far, the majority of the explained variance for outcome in psychotherapy seems to reside outside of the therapy setting, also in children and adolescents [67]. Or, more precisely, results from the interaction between extra-therapeutic factors, specific interventions, common factors, and placebo, and the idea that we can partition the variance is probably an illusion. There is, however, disagreement as to what exactly constitutes extra-therapeutic factors. Some defines it as ‘initial distress’, ‘motivation’, and ‘external support’ [13]; others as ‘individual personal attributes’, such as the ‘personality’, ‘previous life experiences’, ‘current life circumstances’, ‘health status’, ‘hereditary factors’, and ‘any other personal characteristic’ that can negatively or positively impact the outcome of psychotherapy [68]. Others include factors such as ‘faith’, ‘persistence’, supportive family members’, ‘community involvement’, ‘job’, or a ‘crisis situation’ [69, 70]. Notwithstanding the conceptual differences and the absence of a consensus definition, extra-therapeutic factors are in this text defined as ‘occurrences outside of the counselling room’ [70, 71]. Many studies document a strong effect on psychotherapy outcomes of factors outside of what happens in the therapy room (e.g. quality of social networks). Billings and Moos (1985) conducted a 12-month prospective, longitudinal study of 380 adult depressed clients at six in-patient and out-patient clinics and found that a major contributor to outcome was negative home environments and family arguments [72]. In a similar study, Moos (1990) explored the extra-therapeutic effects of stress and social supports on the efficacy of treatment programs on 265 depressed outpatient adult clients, and found that social support and environmental stress together explained a significant portion of the variance in the amelioration of depression [73]. In a recent meta-analysis exploring the effect of non-directive supportive therapy for adult depression, approximately 34% of the variance of the outcome was found to be related to extra-therapeutic factors and 50% to non-specific factors suggesting that the effect of specific factors is limited at best [74]. Although, not explored as thoroughly in adolescent populations, the same results seem to emerge here [67].

Findings from a recent study suggest that actual social support is relevant for the level of social anxiety symptoms in adolescents, indicating that interventions that increase social support may help reduce social anxiety in adolescents [75]. Furthermore, building on Bordin´s [76] pioneering conceptualization of the therapeutic alliance as a relational bond between patient and therapist and agreement on the task and goals for therapy, Friedlander et al. (2006) stressed the importance of how other individuals, beyond the therapeutic relation, affects the process and outcome for psychotherapy. This is also referred to as the *systemic alliance* and comprises two aspects. First, the relational bond and agreement on task and goals *only* between therapist and patient, and second the social impact of other vital relationships in the patient’s life, e.g. friendships, social relationships, romantic relationships, familial relationships etc. [77]. Findings do support the importance of support or lack of support from relationships outside the therapeutic setting, for the outcome in psychotherapy [78–81]. In fact, some therapies for adolescents have their primary focus on modifying the multiple systems surrounding the young person rather than intervening directly with the individual with presenting problems [82, 83]. The importance of extra-therapeutic context is also underscored by failures to replicate the efficacy of particular approaches across international boundaries (not least approaches that major on the modification of ecology) [84, 85].

Hence, the impact of the social context on the therapy process, including the connection one feels with those social relationships, are crucial for understanding the outcome in psychotherapy [86], and a strong body of evidence support the relationship between high ratings of systemic alliance and treatment progress, emphasizing the importance of extra-therapeutic factors in psychotherapy [87].

### How mentalization-based theory can explain the lessons learnt from psychotherapy research: towards a socioecological approach as framework for enhancing treatment outcomes in young people with BPD

In this section we draw on recent advances in mentalization-based theory to try and explain the lessons learnt from general psychotherapy research as described above, and we apply the recent advances in mentalization-based theory to propose a socioecological approach to treatment that may offer a broader and potentially more productive theoretical framework to enhance some of the general factors that explain effectiveness common to all psychotherapy as well as the influence of contextual (extra-therapeutic) factors discussed above.

First, in the previous section we showed that psychotherapy works for the majority of patients in treatment compared to those not in treatment. Mentalization-based theory explains this finding in terms of the assumption that therapy – of any sort – provides a relational context in which learning and behaviour change takes place (Luyten et al., 2020). Thus, the first principle of the socioecological approach we propose for young persons with BPD is that therapy absolutely has to provide a relational context in which the young person’s unique perspective is taken seriously, understood and communicated as such. This will foster the necessary agency that will ensure long-term treatment gains [88, 89]. While facilitating a feeling of being understood in a relational context is a cornerstone of MBT for adults, we highlight this feature as it is particularly important in adolescents, who are dealing with a normative identity crisis [90], and are especially vulnerable to feeling misunderstood and alone [91].

In the previous section we also showed that common factors account for a large part of the variance in treatment outcome, implying that different evidence-based treatment modalities perform equally well (or poorly). This could be, as articulated by mentalization-based theory, that all effective psychotherapy – regardless of orientation—make use of ostensive cueing to increase trust (lowers epistemic hypervigilance) and enhance social learning, which together sets the stage for therapeutic change. Ostensive cues refers to the fact that communication not only transfers information, but also signals that it is intentionally communicated to the recipient [92]. The therapist “puts her mind on the table” to make clear her intentions for communicating a particular piece of information and to signal that the content of her mind is different from the content of the client’s mind (Sharp & Bevington, in press). In so doing, the therapist’s mind becomes less opaque to the client and the client begins to understand why the therapist raises a particular point. The therapist does not speak as an authoritative figure who knows the deficiencies of the patient. Neither does she speak as a cheer leader, blindly cheering on her patient. Instead, she declares what is on her mind in relation to the patient and thereby signals a gap between her understanding of the patient’s mind and what the patient may present. This gap offers a collaborative learning opportunity to the patient. As such, ostensive cues are essential for information to be trusted and activates a pedagogical stance for learning about new information and leaves the recipient with a sense of being acknowledged and understood as an agentive self [93]. Epistemic trust refers to the capacity to identify knowledge conveyed by others as personally relevant and generalizable to other contexts [94], and epistemic hypervigilance is a mode of mistrust obstructing learning to occur. Following this reasoning, it is not as important what *particular* model is presented, but rather that *a* reasonable model is presented. Relatedly, this social learning paradigm of psychotherapy processes also implies that all evidence-based therapies must meet the patient with acknowledgment and curiosity about his/her experiences and perspectives (a mentalizing stance). Indeed, mentalizing has been suggested as a common factor for treatment in general, and of BPD treatment in particular [88, 95–97], because it activates the social context in which the patient functions, not only in the therapy room but also outside of it. This feature of MBT is particularly important in adolescence, which is a developmental stage characterized by increased mistrust as young people move the source of knowledge from parents to peers [98]. Thinking about how an adolescent is making use of learning that takes place inside the therapy room when outside in the world therefore forms a key feature of the socioecological approach we propose here.

The third group of research findings discussed in the previous section collectively suggested the therapeutic alliance to be an important predictor of outcome, perhaps especially so for children and adolescents, and should be expanded to also include the alliance between caretakers (family members) and therapist. This is consistent with a social learning perspective emphasising the importance of a trusted relationship as a natural foundation for learning to take place [99], and forms a central part of a socioecological orientation. Specifically, for young people, their parent’s relationship with the therapist is important and predicts outcome in psychotherapy, underscoring that it is not only the dyadic relationship between therapist and patient that is important. Not only close relationships between the child and its primary caregivers are important in personal development, but also the wider socio-cultural context [100]. This has direct consequences for treatment of BPD, in the sense that a somewhat different treatment approach is warranted, where the broader social life of the patient is targeted more thoroughly [101]. Recently, using data from a treatment study of BPD in adolescence [102], we demonstrated that a fundamental protective factor against drop-out was high levels of trust in parents, stressing that relationships beyond the patient-therapist is important for attendance to psychotherapy and psychotherapy outcome [103].

The fourth class of research surveyed in the previous section showed that therapist characteristics played an important role in outcomes for adults, but appeared less significant for child and adolescent outcomes. Recent socio-evolutionary findings have shown that children are programmed to learn from others [104], and perhaps for this reason, from a very early stage children differentiate between those who make true or false claims, although they may be in general more likely to develop epistemic trust than adults are [105–108]. So, success in the treatment of BPD in adolescence rests perhaps less upon who conducts the therapy and more upon the therapist´s aptitude to create safe relationships where the youngster’s inborn capacity to create and mobilize trust in others can flourish. Put differently, a critical role of the therapist is to activate the socioecological context for the patient. What happens inside the therapy room may be important to initiate change, but sustained change will only come if the socioecological context is activated. This has become a central focus in recent accounts of mentalization-based treatment [109] and has been shown also in the MOBY trial in Australia [110].

The fifth theme discussed in the previous section relates to research on placebo effects in children and adolescents and indicated that placebo effects in children and adolescents in general, do impact outcomes with equal effects as in adult populations. This finding probably covers: 1) higher levels of credulity in children in part because they have no reason to be vigilant, therefore “expecting the best” and trusting those they encounter more readily [92, 111, 112], and 2) on the contrary, adolescents who are known to display more hypervigilance towards information coming from adults [113].

Also, research show that children’s learning capacities are greater compared with adults [114]. Findings suggest that parents play an essential role here, and their role could explain some of the high placebo effects encountered in child and adolescence psychotherapy research. Specifically, research points to observational learning as an explanation [115]. As a consequence, a socioecological focus in the form of including parents in treatment of children and adolescents is essential [116]. Too often, children and adolescents are dropped off for individual sessions with a therapist and parents are not actively included as part of the socio-ecology of their child. Based on these findings, it makes sense to include parents and other important relationships in the youngster’s life to a greater extent as is most often done, as they (can) have a positive impact on treatment outcome. Furthermore, we need to make the most of the learning-potential and trust we encounter in adolescents in the context of psychotherapy.

The sixth group of findings discussed in the previous section concerned the use of feedback tools in psychotherapy. Here the available research with adolescents demonstrated fewer convincing results compared to adults. Research showed that applying feedback-informed measures in adult population enhanced the therapeutic alliance [117]. The feedback informed approach is not only about measures and tools, but about facilitating ‘connection’ and assisting the establishments of trusting relationships. Using feedback informed methods also conveys a feeling of agency to the patient [118]. It is about creating a collaborative stance, a “we-mode” [119] that is central to treatment [120].

Again, these are components that already form a key component of more recent formulations in the mentalization-based approach of social learning (Luyten et al., 2020), where it is argued that a sense of control and an acknowledgement of agency in the patient are vital for positive outcome in psychotherapy. In the MBT approach it is argued that psychotherapy centres around three learning systems (see [121]. In learning system 1, a general treatment model is presented for the patient, and that works as ostensive cueing leaving the patient trustful toward towards therapy. If that includes a feedback informed tool that acknowledges the active participation of the patient, that would probably contribute to a better therapy process. However, the feedback approach does not seem to work as well in adolescent populations as compared to adults for reducing symptoms [52]. There is no obvious reason for this finding [52], and one can only speculate if the ‘methods’ and ‘techniques associated with Feedback Informed Treatment (FIT) stand in the way for good treatment in adolescents. Some research indicates that the FIT approach in adolescent psychotherapy could work as a mediating factor through the positive change in parents expectations working with FIT [51], but this is yet to be robustly tested. Generally, adolescents are characterized by higher levels of epistemic hypervigilance, suggested as a normative state in this period [122], and FIT may represent a “boring” or “trivial” initiative for youngsters. Hence, FIT may not be the appropriate tool to enhance psychotherapy outcome in adolescents with BPD, given the high negativism towards adults and that they are broad to therapy with issues that their parents think need to be treated.

The seventh theme of research discussed in the previous section showed that for adolescents, the importance of manualized treatment compared to non-manualized treatments remains unclear. Although for example MBT is a structured treatment approach, aiming to deliver coherent, consistent and continuous treatment [109], it is fundamental that the perspective of the patient is acknowledged while curiously exploring how she/he comprehends the world. So, when psychotherapy is carried out in a structured fashion with a mentalizing stance, the exact therapeutic modality is less important than is maintaining the mentalizing stance. Again, we see the socioecological focus take precedence over the technique with a clear prioritization of fostering connectedness even if that means that a diversion from the manual is undertaken. Important however, will the fact that therapist mark diversion from the manual in order to articulate the dilemma presenting itself to the client.

The final group of findings discussed in the previous section highlighted the role of extra-therapeutic factors, specifically social support outside of the patient-therapist relationship, and the importance of these factors for outcomes. This general finding forms the backbone of the socioecological approach in work with adolescents, because adolescents are still very much embedded in a social system over which, on the one hand they have little control, and on the other hand offers critical collaborative opportunities to enhance treatment outcomes. We will further elaborate on with a case-example below in which we highlight the adaptations we suggest for further improvement of treatment in adolescent BPD. These suggested adaptations are summarized in Table 1 and demonstrated in the case example below. In sum, as we hope to show, incorporating a socioecological approach entails working *in* and *with* the young person’s social world, prior to and/or simultaneously with therapy. Before turning to the case-illustration, it is particular important to emphasize that apart from indications displaying mentalizing as a common underlying factor for different treatment modalities for BPD (dialectical behaviour therapy, schema-focused therapy, transference-focussed psychotherapy and MBT) [96, 123], there is not enough evidence to support mentalizing as *the* driving mechanism in psychotherapy for BPD. Also, we stress that MBT is necessarily not the best treatment approach to enhance mentalizing, and is by no means superior to other treatment approaches for BPD in adolescence [124]. For example, dialectical behaviour therapy, has shown very good and rapid results in reducing self-harm in adolescents with BPD features [125, 126].

**Table 1:** Overview of socioecological interventions and instruments used in the treatment of BPD in adolescents

Pretreatment network meetings	Three to four network meetings held prior to therapy is initiated. All persons involved in the patient´s life and considered essential for the development are invited. The aim is to register obstacles for a positive development, including barriers to therapy, school attendance, leisure activities, meeting with peers etc. A second aim is to engage the development of the system case-formulation and the system attachment map
System case-formulation	The system case-formulation is established as a result of pretreatment meetings and ongoing network meetings, formulating how specific elements contribute to the patient´s current state, both barriers as well as resources
System attachment map	The intention with the system attachment map is to map and describe the most vital attachment patterns in the patient ´s life to other people outside of therapy, and to clarify what potential risk of conflict these patterns could cause for the patient
NET-AIM	The NET-AIM is a self-report instrument capable of keeping track of and evaluating the network interventions and meetings to secure common ground and gauge network alliances and network trust. All network participants answer the questions and it is used as a common tool in the network to evaluate interventions and collaboration
Network meetings	Network meetings are essential and is implemented with almost the same frequency as therapy. In those settings the system case-formulation and the system attachment map are developed at revised as things change. The network meetings are monitored using the NET-AIM

## Case presentation

### Improving and expanding effective treatment for adolescent BPD through a socioecological approach – a case illustration

In the following case illustration, we highlight how taking a more socio-ecologically informed approach to the treatment of adolescent BPD was carried out in a case with a 16-year-old girl with BPD. The patient was seen and treated at an outpatient child and adolescent psychiatric clinic in Denmark, where the first author works. Names and sensitive information have been changed to ensure anonymity.

Jennifer, a 16-year-old girl not previously known in the psychiatric system, was referred for psychiatric assessment to an outpatient psychiatric clinic due to intense interpersonal conflicts, major mood changes, self-harm, many aggressive outburst, feelings of insecurity in social situations and a prevailing feeling of emptiness. After the initial intake session in the clinic with Jennifer and her mother, it was decided to assess if Jennifer´s problems could be diagnosed as PD. Consistent with the socioecological approach, we also included her parents in the assessment, and adapted the questions from the Structural Clinical Interview for DSM-5 Personality Disorders (SCID-5-PD) [127] to include both adolescent and parent report. With permission, we also collected information from other sources, including her school, a club she was attending and her social worker. This was carried out to ensure the most adequate and valid assessment and to potentially rule out differential diagnoses, but also to signal to the social network surrounding Jennifer that we considered them a fundamental part of both the assessment and treatment procedures. Jennifer was diagnosed with BPD and Avoidant PD and referred to a mentalization-based treatment program, comprising individual MBT session in combination with family MBT therapy. The program is based on a socioecological approach. The program is not set to last for a specific time period, for the simple reason that we do not have data supporting such a specific recommendation as yet. The treatment-program with Jennifer lasted approximately 14 months. Furthermore, we did not settle on specific clinical criteria in terms of problem reduction as a mean to determine when to terminate treatment. Rather, we assessed her quality of life or functional level and decided beforehand to end treatment when she was able to engage in, what appeared to be, a more or less ordinary teenage life. Before engaging therapy three pre-treatment network meetings were held to clarify resources and obstacles in the social system, not only for Jennifer´s individual therapy (e.g. attending therapy), but for a range of aspects she considered important in her life (school, leisure activities, peer-relations, parent interaction). Jennifer had one friend whom she shared the joy of horse-riding. Jennifer was unable to catch up with that friend, because Jennifer and her mother recently moved and the travel distance in train was overwhelming. Also, she was not comfortable in her class at the new school and often skipped school, because she felt lonely. A third and important obstacle was that she had a volunteer job at a pet-store at the same time as her individual therapy was scheduled. Her father refused to participate in family therapy, he claimed he was not her real farther, and that hurt Jennifer. Finally, the new location she moved to with her mother was very isolated and had very few connecting bus-lines making it difficult to meet with peers. Thus, before initiating the MBT-therapy, and to clearly acknowledge contextual issues in Jennifer´s life, meetings were held with the school, Jennifer´s social counselor, employees from the social club she was attending, and a social worker who was coupled to the family. It was arranged that that the social worker would meet with Jennifer three times a week, and drive her to the riding course once a week. The social worker would also try to support Jennifer in catching up with other peers and help her with transportation once a week to the club. The school agreed to support a better integration of Jennifer in the class and allocated six hours weekly support from a teacher she felt safe with, who would help her especially in the mornings where Jennifer had a hard time entering the class. It was negotiated with Jennifer that every second time she should attend therapy, her work schedule would be re-arranged and then the next therapy-session would be moved so no changes in work plans had to be made. Despite attempts to engage her farther in MBT family therapy, he continuously denied to participate.

The three pre-treatment meetings were held before individual and family MBT were initiated to ensure support from the broader social context and to convey the message to Jennifer and the network that Jennifer´s issues were not just intrinsic deficits in her personality structure, but also a matter of socioecological factors. As an essential part of the final pre-treatment meeting a “system case-formulation” was presented. The individual case-formulation is known in most therapeutic paradigms as a central feature for a structured treatment [128], linking the patient´s past, present and future with current issues and setting therapeutic goals to work with. The system case-formulation is a supplement to the individual case-formulation – signalling and emphasising a “systemic responsibility/role” for the current problems encountered, as opposed to an intrinsic focus on the youngster’s problems. It also sets specific goals for other people working with the patient. In the system case-formulation specific factors relevant for the patient’s well-being are noticed, and goals are set up, defining who and what should be done do accomplish these goals. It is often challenges linked to the patient´s life, but with an acknowledgment of those challenges having an association to other people’s actions and being dependent upon collaboration with others to be resolved. The outcome of the system case-formulation in Jennifer´s case was a specific focus on the problems she encountered in school and an agreed assignment that the social counsellor together with the school had to facilitate and support her in this context.

After the final pre-treatment meeting, Jennifer stated: *“This is the first time anyone acknowledges that it is not just me who are wrong, but things around me are a mesh too, what a relief… but why did it take 16 years for you guys to realize that?”*. Another outcome of the pre-treatment meetings was the development of a “system attachment map” (a supplement to the “interpersonal passport” used in MBT therapy, see [89]. The idea is to map and describe the most vital attachment patterns in the patient ´s life to other people outside of therapy, and to clarify what potential risk of conflict these patterns could cause. In MBT therapy, there is a continuous focus on the relationship with the therapist, the here-and-now relationship. However, many therapists seem to miss the opportunity to work in and with the current relation, hence a new feature in MBT therapy is to be aware of the attachment pattern between the patient and therapist and explicitly have that in mind during therapy [89]. Our idea was to broaden that approach and include attachment patterns between the patient and essential key-figures in the larger social system also.

This approach was informative, at least in this case, and specifically for Jennifer´s social worker and her teacher. It also clarified how the key-figures around Jennifer sometimes contributed to an undesirable relationship. Jennifer reached new insights: *“aha, that is right, I am a bit ambivalent about you Carl (her social worker) and yes, I totally mistrust Vita (schoolteacher)”*. The network agreed to work explicitly with and maintain focus on both the system case-formulation, including the goals set-up here, as well as having the system attachment map present when co-workers from the network interacted with Jennifer.

The family therapy session, unfortunately only including Jennifer and her mother and not her father, had a specific focus on their relationship at home characterized by intense and loud arguments, and often resulting in Jennifer self-harming. Again, the MBT family therapy sessions had an explicit focus on understanding the dynamics between the two of them, and not interpreting the chaotic interpersonal situations as a result of Jennifer´s PDs alone, but rather how misunderstandings, emotional arousal and basic mistrust from both parts often caused the exhausting conflicts. The individual MBT therapy was delivered ad modum the MBT manual [89], with adaptations made specifically for the developmental challenges encountered in adolescence [98].

There was substantial improvement in Jennifer´s attendance at school. According to the teachers the focus created with the system case-formulation was very helpful for them and helped them maintain focus and not lose track on their task. Jennifer explained how the school teacher facilitated new social interactions with other peers in the school yard: *“she kind of made me a part of that soccer group without making it awkward – I hardly noticed that suddenly I was part of them girls playing in every break”*, and further: *“I felt connected with them and no longer saw myself as the odd girl in the corner—Vita (the school teacher) really made a difference there”.*

The approach with the system attachment map in regard to Carl (the social worker) seemed to work out well. Jennifer felt ambivalent in her relationship with Carl, who was employed to support and help her with various things after school. *“Well, Carl is sometimes an okay guy, and other times he is really, really annoying, I think he talk too much with mum, telling her all the stuff I tell him not to report”*, Jennifer explained in the beginning. Later she reported that having had the focus on the ambivalence in their relationship helped her gain insight into her own contributions to the ambivalence. *“It was Carl who almost every time we met asked me about our relationship… that kind of made me think of the whole thing and my doubts in him”*. On the other hand, Carl also explained how he got an understanding of his parts in that: *“Well, after keeping such a strict focus on the relationship between Jennifer and I, I started reflecting about my own role in her feelings of mistrust and doubts towards me, and that made me understand that I actually played an active role in that every time I met with Jennifer´s mother”*.

The system case-formulation also helped both the therapist as well as the broader network to keep track on risk-factors in Jennifer´s life. Earlier she was part of a small gang of girls harassing and intimating random people on the street. However, with that specific risk-factor mentioned in the system case-formulation and Carl (the social-worker) now being aware of that risk, Jennifer and him was capable of keeping Jennifer out of the group despite powerful attempts from various of the girls from the group to get her back in. *“If Carl had not dared to mention that thing with the gang, I would probably have been part of it again, but the fact that we agreed to work on it, and found other things to do, did that I stayed away from them”.*

When Jennifer was around 16-years old and before she was referred to the psychiatric unit, she had sexual relationships with various men way older than herself. Those encounters took place at a hotel where she worked as a bellboy. That was also noticed in the system case-formulation since Jennifer was very dependent on the money she earned. As an alternative to the job at the hotel, Jennifer´s social counselor helped her find another job with less risk for set-backs, and at the same time her sexual promiscuous behavior was worked with in her individual MBT therapy. Jennifer mentioned: *“It was so important I got away from the hotel and got a chance to work with that “old-men”-issue in my therapy, I wouldn´t have accomplished it if I was still working at the hotel, I think”.*

A fundamental result of introducing the system attachment map and the marking of the ambivalence in the relationship between Jennifer and Carl (social worker), was that it helped Jennifer gain a better relationship with her mother. Basically, they did not trust each other, but as a result of the work Jennifer and Carl carried out, Jennifer started trusting her mother again. *“When I first learned to trust Carl, it was much easier to believe in what my mother told me”*, hence a re-establishment of epistemic trust [94].

In terms of keeping the network interventions aligned we used an instrument called NET-AIM capable of keeping track of and evaluating the network interventions and meetings to secure a common ground and focus and gauge network alliances and network trust (Walløe & Lock, in press). This instrument has been developed to monitor and align interventions where various people from different sectors are involved in a treatment of a patient. First of all, to avoid or at least decrease misunderstandings, second to ensure that those involved have a common understanding of the problems and for the implemented interventions.

Generally, the frequency of the different interventions within this socioecological framework are as follows: three to four pre-treatment network meetings before therapy is engaged (Family therapy and individual therapy). In those meetings the basis for both the system case-formulation and the system attachment map are formed. Furthermore, major obstacles for attendance to therapy and other important things in the patient´s life are identified and a plan is made concerning how to deal with those issues. Network meetings are held on a continued basis (at least once a month) with all members of the network. MBT family therapy is once a month and individual MBT twice a month. Interventions are guided by the principles of the clinical staging model, and applied as a heuristic strategy and an alternative to the conventional categorical classification system. The clinical staging model offer better options to evaluate dimensional severity of symptomatology [129], including BPD, and to design interventions according to severity and the stage of the BPD syndrome [130, 131].

After end of treatment we found that Jennifer improved her functioning in a range of areas. First of all, her capacities for relationships improved significantly, and she was better able to establish and maintain stable connections with young people, and felt less anxious before catching up with friends. During the treatment program Jennifer´s ability to regulate emotions improved and she displayed fewer outbursts of anger. She acquired new relationships, including two new friends through her work. She also spent more time at the club, engaging relatively fully in activities with other young people. Her descriptions of her interpersonal experiences suggested that she was experiencing feelings in a more nuanced way and that this helped her specifically in managing her relationship with her mother. Although the socioecological treatment approach alleviated many of the issues Jennifer dealt with, she did not fully recover from all her challenges, including those related to BPD. She still faced issues with frequent self-harming, specifically when feeling lonely, and although her sense of self (or personality) was enhanced by the intervention, she sometimes still experienced identity diffusion and serious doubts about her identity. Although, the socioecological framework provided a framework for both managing risk factors as well as targeting points for therapy, Jennifer nevertheless often felt restless and sometimes missed out on therapy sessions. After the end of treatment, she maintained the work with Carl and was to some degree still dependent on weekly support from him. Nevertheless, and over the course of the treatment, we did see a substantial and general enhancement in Jennifer’s quality of life and in her capacities to manage interpersonal situations as well as an overall improvement in her level of functioning.

## Discussion and conclusions

The aim of presenting a socioecological approach to the treatment of BPD in adolescents grew out of a paucity of evidence for what treatment work best for adolescents with BPD and a somewhat limited effect of the current treatments available. The extant research literature is inconclusive on those matters. We then sought inspiration in the evidence from general psychotherapy research to find that a great proportion of the variance for outcome in psychotherapy is explained by extra-therapeutic factors. Inspired by MBT theory, specifically the social learning theories, we devised a socioecological approach to the treatment of BPD in adolescents.

The socioecological approach to the treatment of BPD in adolescents as outlined in this paper, including the interventions proposed in the case-illustration above, may seem excessive in terms the resources expanded, i.e. gathering multiple people together relatively frequently. On the other hand, research findings indicate the very substantial economic burden of BPD in adulthood [132, 133] and calls for early intervention with young people with BPD [130] to prevent the development of later severe personality pathology. Thus, although the socioecological treatment approach, as presented here, seems to require much personnel and time, an early focussed effort to reimagine a young life with all its complex network of relationships may pay off in the long run [110]. Also, as the approach is built on cooperation between different professional sectors with a common learning process being established during the meetings. Hence, a time-consuming introduction to the treatment approach is not required for the clinician prior to engaging in the delivery of the treatment. The idea is that learning occurs ‘as we go along’, and that a more experienced clinician works closely with less experienced clinicians creating a natural process of dissemination and knowledge transfer. This is very much in line with the dissemination approach taken in the Adapted Mentalisation-Based Integrative Treatment (AMBIT) model, also originally created for young people, where dissemination and implementation is a forthgoing dynamic process [134]. We also note that currently there is no shorter-term, and effective, approach available for addressing personality challenges in young people. We propose a move away from a reactive, purely individual therapeutic approach, to investing in a more systemic, proactive and sustained approach to scaffold the development of personality in young people.

Furthermore, the socioecological approach also seems to deviate from the more conventional and traditional dyadic therapeutic setting where BPD patients often are treated. However, as we interpreted the evidence from general psychotherapy research, it was clear that a substantial amount of variance in psychotherapy outcome stems from extra-therapeutic factors. Also, in line with research presented here showing that the quality of real-world relationships, not just the patient-therapist alliance, is of great significance for the prognosis and outcome. Results do indeed support the impact of help from relationships outside the therapeutic setting for the outcome in psychotherapy [78–81]. Hence, the impact of broader social factors on the therapy process, and the connection one feels with those social relationships are crucial for the outcome in psychotherapy [86]. While other socioecological approaches have been developed for conduct disorder [135] and hard-to-reach youth [136], these approaches have not been extended to youth with personality pathology. Also, the socioecological treatment approach to BPD could very well be suited and extended further, to include day treatment and/or inpatient approaches to treatment. Specifically, treatment of BPD in hospital settings has, contrary to earlier clinical findings, shown to benefit patients and contributing to an increase in recovery [137, 138]. Including inpatient or day-treatment as an aspect of a socioecological approach could potentially support, and may even be critical in the translation of intervention strategies from the treatment system to the adolescent’s everyday life. Moreover, group therapy may also offer an important additional context for the translation of intervention effects. While efficacy of MBT group therapy in adolescents with BPD is still to be established [102, 139], group therapy in adults have proved to be an important venue for generalizing individual therapeutic effects [140].

The socioecological approach seems specifically suited for adolescents due to the normative developmental challenges they encounter [98], and the fact that they are more dependent on adults and family in a range of areas compared to adults. However, the approach might also be feasible with adults, and is consistent with the Good Psychiatric Management [141] suggesting that a major focus of therapy should be “getting a life”. Moreover, as long as we do not have strong and convincing evidence for the current treatment programs of BPD in adolescents [8, 10], and with the research supporting interventions targeted at the broader social context recent, we would conjecture that it is feasible to pursue the socioecological approach when designing treatment programs for adolescents with BPD.

### Limitations

There are several limitations to the approach suggested here. First of all, we conducted a selective review of both the research literature on BPD in adolescents, and of factors important for general psychotherapy research. We are aware that the list of factors important for psychotherapy listed here are not complete. Second, we cannot directly transfer all results found to be essential in general psychotherapy research, to treatment of BPD in adolescents. However, we included that framework, as argued earlier, since the specific research on the treatment of BPD in adolescents is incomplete. In addition, we included ideas from MBT theory, and those thoughts are only partly confirmed in the empirical literature [109]. Relatedly, the socioecological approach to intervention for youth BPD must be evaluated empirically in the context of a randomized controlled design. For future dissemination of this approach, it will be critical to show that the addition of the socioecological approach beyond the therapist-client dyad improves outcomes.

Notwithstanding, these limitations, we have proposed a somewhat different framework for clinicians and researches that can be used in the treatment of BPD in adolescents. We conclude our paper by giving Jennifer the final word: “…*without those weird roundtable meetings with all those people, and you guys helping me out there where I actually live, I would never have come so far…”.*

## Data Availability

No data used in the current paper.
